# Telemonitoring with an implantable loop recorder in outpatient heart failure care: One year follow-up report from a prospective observational Dutch multicentre study

**DOI:** 10.1007/s12471-018-1198-x

**Published:** 2018-12-03

**Authors:** R. S. S. Kort, Y. S. Tuininga, H. A. Bosker, M. Janssen, R. Tukkie

**Affiliations:** 1 Department of Cardiology, Spaarne Gasthuis, Haarlem, The Netherlands; 2 Department of Cardiology, Deventer Hospital, Deventer, The Netherlands; 3 Department of Cardiology, Rijnstate Hospital, Arnhem, The Netherlands

**Keywords:** Heart failure, Atrial fibrillation, Telemedicine, Ambulatory care

## Abstract

**Introduction:**

In the care of heart failure patients, telemonitoring is receiving growing attention. The main purpose of this study was to determine the effect of continuous telemonitoring with an implantable loop recorder (ILR, Reveal XT), a novel strategy in the management of stable heart failure patients without a cardiac implantable device. Furthermore, little is known about the incidence of subclinical arrhythmias in this specific group of patients.

**Materials and Methods:**

Stable heart failure patients, New York Heart Association Class II and III, without recent hospitalisation or upcoming intervention, were included. After implantation of the ILR there was regular contact with the research nurse on a pre-specified basis. Clinic visits and telephonic interviews were alternated for a minimum of 1 year. Parallel visits to their treating physician continued according to standard care. The treating physician was blinded for the ILR findings, accept for pre-specified, significant arrhythmic events.

**Results:**

Thirty patients were included and followed for a median duration of 12 months. In 13 patients, data from the loop recorder led to therapeutic changes. One patient received a pacemaker. Eight patients developed atrial fibrillation, all subclinical, with a mean burden of 65.8 ± 173.2 min/day.

**Conclusion:**

The use of an ILR could potentially impact patient management. Additional study is needed in different patient populations (e. g. higher risk groups) to assess if an ILR could also impact on endpoints such as heart failure hospitalisation.

## What’s new?

- To the best of our knowledge, this study is first to report on the use of implantable loop recorders for heart failure management.
- Continuous monitoring detected arrhythmic events that led to therapeutic changes in 13 patients. These events were not detected by routine visits to the cardiologist.
- The use of an implantable loop recorder could potentially impact patient management, but additional study is needed.

## Introduction

Heart failure is affecting an increasing number of people worldwide and is associated with substantial morbidity and mortality, a high risk of developing arrhythmias, hospitalisation, and thromboembolic events [1]. The occurrence of atrial fibrillation (AF) predisposes these patients to embolic events and worsening heart failure [2]. Over the last years, telemonitoring has received growing attention in different fields of medicine. Several telemonitoring entities can be distinguished ranging from telehomecare using (semi) structured telephonic assessments, via telemonitoring with transfer of physiological data (such as weight, blood pressure, and heart rate) to remote monitoring in cardiac device patients which adds data such as heart rate variability, atrial tachyarrhythmia detection, patient activity and thoracic fluid accumulation. New invasive technologies are currently being tested measuring, for example, pulmonary artery pressure [3, 4]. These parameters are recorded with the purpose of positively influencing heart failure outcome, increasing patient participation and early adjusting therapy in case of heart failure deterioration.

Multiple studies have investigated the effect of telemonitoring on reducing hospitalisation and mortality [5–9]. Results of these studies have been contradictory. The meta-analysis by Inglis et al. suggested a positive effect of telemonitoring on all-cause mortality and hospitalisation [10]. However, these findings have recently been challenged by two large multicentre randomised trials. The Tele-HF study, using structured telephonic interviews, showed no improvement of the primary endpoint (hospital readmission within 30 days for any cause), compared with standard care [11]. Also, the TIM-HF trial requiring daily self-assessments significantly reduced all-cause mortality in only a selected group of patients with a good mental state [8, 12, 13].

A large proportion of systolic heart failure patients receive treatment with an implantable cardioverter-defibrillator or cardiac resynchronisation therapy device (pacemaker or defibrillator). These devices offer the chance to continuously monitor physiological and technical data. In the IN-TIME trial, telemonitoring significantly reduced all-cause mortality and it was hypothesised that three mechanisms contributed in parallel to this improved clinical outcome [5]. The three proposed mechanisms were early detection of tachyarrhythmias, early recognition of suboptimal device function and raised patient awareness. In heart failure patients without a current indication for a cardiac implantable device, continuous data collection on early detection of tachyarrhythmia could also be achieved by an implantable loop recorder (ILR). No studies have been performed using an ILR for the sole purpose of early arrhythmia detection in heart failure patients. We anticipated that an ILR could be useful to fulfil this gap.

The main purpose of this study was to determine the usefulness of telemonitoring, with an ILR as a novel strategy in the management of stable heart failure patients without a cardiac implantable device and without oral anticoagulant (OAC) therapy. Secondarily, little is known about the incidence of subclinical arrhythmias in this specific group of stable heart failure patients. In this paper, we report the 1 year follow-up data.

## Materials and Methods

### Study design

This study was a prospective, observational multicentre study performed in three large Dutch non-academic teaching hospitals (trial registration: NCT01366703). Patients were recruited between July 2011 and April 2014 and were included after giving written informed consent. Minimal follow-up duration was 1 year. Because of the nature of the study (descriptive without statistical assumption because of lack of previous data) no formal power calculation was done to determine the sample size. A patient sample of 30 was considered reasonable. The study was approved by the Medical Research Ethics Committee of Noord-Holland.

### Inclusion and exclusion criteria

Patients with stable systolic heart failure (ejection fraction 35–45%) or diastolic heart failure (ejection fraction > 45% and echocardiographic signs of impaired diastology or NT-proBNP* >*250 ng/l*) *were included. Stable was defined as a New York Heart Association (NYHA) Class II or III, without hospitalisation in the last 3 months or a scheduled intervention in the coming 3 months. Patients with a pacemaker/ICD or an indication for such a device were excluded. Since we sought possible therapeutic implications from continuous monitoring, patients with known AF, a virtual CHA_2_DS_2_-VASc of 1 or less or already on an OAC were excluded.

### Reveal XT implantation

After inclusion, the implantable cardiac monitor Reveal XT (Medtronic Inc., Minneapolis, USA) was inserted in a routine fashion. Patients were placed on home monitoring with Carelink and the following alerts activated: ventricular tachycardia, asystole of longer than 3 seconds, bradycardia and AF. All Carelink alerts were checked daily by a device technician during in office hours on weekdays.

### Follow-up

Patients were followed in a parallel fashion. First, they were seen at the clinic by a research nurse, experienced in heart failure trials, at intake, 6 and 12 months, with additional structured telephonic interviews at month 3 and 9. The research nurse was also responsible for careful case report form (CRF) compliance and CRF documentation, collection of the pre-specified arrhythmic events in the CRF and distribution of the pre-specified arrhythmic events to the treating physician (see later). The ILR was interrogated before visiting the research nurse at month 6 and 12. Second, in parallel, patients were seen by their own treating physician the same day at month 6 and 12. Minimal follow-up was 1 year. Their treating physician was unaware of the ILR findings except for several pre-specified events. Pre-specified events were asystolic episodes of 3 seconds or more, AF or other supraventricular arrhythmia longer than 6 minutes, non-sustained ventricular tachycardia or second/third degree AV block of any duration. If a pre-specified event occurred (detected at a routine visit to the technician or by a home-monitoring alert), the device technician contacted the research nurse who then took appropriate action (CRF update and informing the treating cardiologist). Therapeutic interventions (whether based on a clinical event or after a pre-specified event from the ILR) were made at the discretion of the treating physician.

### Analysis

All data were processed using IBM SPSS 20.0. Continuous data are expressed as mean ± standard deviation, discrete data are expressed as frequency and percentage.

Data retrieved from Carelink were additionally processed at Medtronic Bakken Research (Heerlen; the Netherlands) to obtain aggregated data on arrhythmic events and AF burden, which were exported to an excel sheet. All event tracings were assessed by an experienced device cardiologist (RT).

## Results

### Patient demographics

Baseline characteristics are presented in Tab. 1. Between July 2011 and April 2014, 30 patients were enrolled and received an ILR. The mean age was 71.8 ± 8.8 years. The majority were female (56.7%). At baseline, 25 patients were in NYHA functional class II and 5 in class III. The mean ejection fraction was 44.5 ± 7.8 and 80% of the patients had systolic heart failure.

**Table 1:** Baseline characteristics

age (years)	72 ± 8.8
men	13 (43.3)
weight (kg)	78.3 ± 16
LVEF	44.5 ± 7.8
*NYHA*	
II	25 (83.3)
III	5 (16.7)
*heart failure duration*	
<2 months	4 (13.3)
<3 months	4 (13.3)
<6 months	2 (6.7)
>6 months	20 (66.7)
*medical history*	
coronary artery disease	11 (36.7)
revascularisation (PCI and/or CABG)	9 (30)
valvular heart disease	2 (6.7)
diabetes mellitus	9 (30)
hypertension	19 (63.3)
peripheral artery disease	4 (13.3)
renal insufficiency (GFR <60 ml/min/1.73 m^2^)	6 (20)
*medication*	
beta blocker	25 (83.3)
ACEi/ARBs	27 (90)
antiplatelet therapy	18 (60)
calcium antagonist	5 (16.7)
antiarrhythmics	1 (3.3)
diuretics	18 (60)
nitrates	4 (13.3)

All characteristics are reported as frequencies (percentage), except for age, weight and left ventricular ejection fraction, which are reported as mean ± standard deviation

*ACEi* angiotensin-converting-enzyme inhibitor, *ARBs* angiotensin II receptor blockers, *CABG* coronary artery bypass grafting, *GFR* glomerular filtration rate, *LVEF* left ventricular ejection fraction, *NYHA* New York Heart Association classification, *PCI* percutaneous coronary intervention

### Follow-up

Median follow-up was 12 months. No patients were lost to follow-up. Two ILRs were removed, at 2 and 12 months, due to local discomfort. One patient had a superficial wound infection. Three patients died during follow-up. The first patient with severe triple vessel disease and inoperable left main coronary stenosis died of acute cardiogenic shock within 1 month after ILR implantation. During this month, no events were recorded by the ILR. The second patient died due to a ruptured abdominal aortic aneurysm within 1 month after inclusion, the ILR data were lost. The last patient died in his sleep, no rhythm or conduction disorders were recorded by the ILR. No patients were admitted to the hospital for worsening heart failure.

### Detection of pre-specified events

**Fig. 1:**
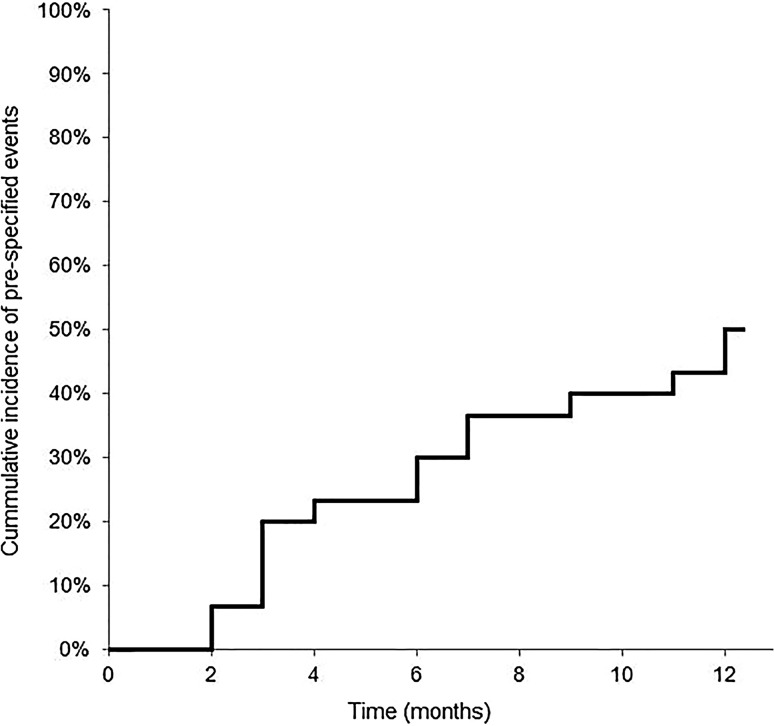
Kaplan-Meier plot: Time to a pre-specified event

In 15 patients (50%) pre-specified events were detected (Tab. 2). The mean time to a pre-specified event was 6.0 ± 3.6 months (Fig. 1). AF was recorded in 8 patients (26.7%). ILR data led to therapeutic changes in 13 patients (43.3%). The following therapeutic changes were implemented: pacemaker implant in 1, institution of OAC in 8 (vitamin K antagonists in 7, direct-acting oral anticoagulants in 1) and adjustments of beta-blocker dose in 6 patients. Of the pre-specified ILR events, none was detected by the routine clinic visits to the cardiologist.

**Table 2:** Overview of detected pre-specified events and actions taken

sex	age	heart failure	event	action
female	81	HFREF	bradycardia/NSVT	none
female	68	HFREF	asystole	dose beta blocker changed
male	65	HFREF	atrial fibrillation	dose beta blocker changed/OAC started
male	78	HFREF	asystole	none
male	86	HFPEF	bradycardia/Atrial fibrillation	OAC started
male	71	HFREF	atrial fibrillation	OAC started
female	77	HFREF	SVT	dose beta blocker changed
female	74	HFREF	atrial fibrillation	OAC started
male	59	HFREF	atrial fibrillation	OAC started
female	74	HFREF	asystole	dose beta blocker changed
female	65	HFREF	atrial fibrillation	dose beta blocker changed/OAC started
male	62	HFREF	atrial fibrillation	OAC started
male	71	HFREF	atrial fibrillation	OAC started
female	71	HFPEF	bradycardia	beta blocker stopped
female	80	HFPEF	AV block	pacemaker implanted

*HFPEF* heart failure with preserved ejection fraction, *HFREF* heart failure with reduced ejection fraction, *NSVT* non-sustained ventricular tachycardia, *OAC* oral anticoagulant, *SVT* supraventricular tachycardia.

### Atrial fibrillation detection

In 20 patients AF was detected by the ILR, 12 were considered false-positive after expert adjudication. Therefore 8 patients were diagnosed with true AF of >6 min. By increasing the minimal duration of AF detection to >60 min, AF was detected in 13 patients by the ILR, 6 were labelled false-positive after expert adjudication leaving 7 patients with true AF of >60 min. The mean AF burden was 65.8 ± 173.2 min/day.

## Discussion

This study is the first report on ILR use, solely for telemonitoring purpose in stable moderately severe heart failure patients. Our results show that continuous monitoring of heart failure patients with an ILR detects more arrhythmic events than standard care and led to therapeutic changes in 13 patients.

The most frequently recorded pre-specified event was AF, detected in 27% of patients, all subclinical. This percentage is in the range of previously reported prevalence of AF in heart failure [14, 15]. This finding also corresponds with the conclusion of the IN-TIME trial, where atrial tachyarrhythmias most often led to unplanned patient contacts [5]. Compared with the IN-TIME study, our patient population differed in several ways. First, our patients did not have a previous indication for a cardiac implantable device, second the mean ejection fraction in the IN-TIME population was much lower (mean 26%) and third 25% of the patients in the IN-TIME trial were already known to have AF. Despite these differences, also in our patient population atrial tachyarrhythmias were prevalent, even more prevalent than might be expected for this patient cohort. There were no hospitalisations for heart failure during follow-up. This can be explained by the patient population under investigation, clinically stable and with a relatively high mean ejection fraction. In further research the study population can be expanded by including less stable patients, for instance after hospital discharge for acute/worsening heart failure to judge the impact of early arrhythmia detection on heart failure hospitalisations.

The detection of subclinical AF is of clinical importance. The benefit of anticoagulant in subclinical AF is proven, but there is no consensus on the duration of AF related to the risk of ischaemic stroke. Boriani et al. demonstrated that the AF burden is an independent predictor for ischaemic stroke [16]. Of the pre-specified durations (5 minutes, 1, 6, 12, and 23 hours) 1 hour was associated with the highest hazard ratio. However, Healey et al. described an already increased risk of stroke for AF duration of ≥6 min [17]*.* Therefore, we reported on the analysis with cut-off points of both 6 and 60 minutes. Twelve patients were labelled false-positively as AF patients by the ILR. If corrected for 60 min, only 6 patients remain labelled false-positive for AF. Podd et al. previously reported on the sensitivity and specificity for detection of AF of the Reveal XT at 79% and 66% respectively [18]. To strengthen the reliability of automated detection algorithms and to reduce the workload of hospital staff, the specificity of the AF detection algorithms should be improved.

### Limitations

Firstly, therapy change was left to clinical judgment in our study. The institution of OACs in case of subclinical AF detected by cardiac implantable devices is under debate [19]. The impact of OAC therapy (including direct anticoagulants) in subclinical device-detected AF is not yet proven and currently being studied in the NOAH-AF trial (NCT02618577).

And second, the study was not randomised but to a certain extent the patients served as their own control group, because treating physicians were mainly blinded to the ILR findings. Although pre-specified findings were reported to the cardiologist, none of these were detected by the routine visits of standard care. Physicians might have been biased knowing that they would be provided with the ILR findings should these occur. Also, this finding does not necessarily imply that the pre-specified events would not be detected clinically later on, but telemonitoring has proven to detect clinically relevant events much earlier. In the Trust trial, the median time to evaluation was <2 days in the home monitoring group compared with 36 days in patients without home monitoring [20]. These findings were reproduced in the Connect trial were the median time from a clinical event to clinical decision per patient was reduced from 22 days in the in-office arm without home monitoring to 4.6 days in patient on remote monitoring [21].

Finally, only 30 patients were included over several years. No one reason can be given for this slow inclusion; however, precise data are missing since no screening log was kept. During the trial inclusion period, several competing heart failure trials were running in our departments. Also, some suitable candidates were unwilling to undergo an ILR implant.

## Conclusion and future implications

We conclude that continuous monitoring of heart failure patients with an ILR detects more arrhythmic events and leads to a relevant number of therapeutic changes compared with standard care. Devices such as an ILR add considerable initial costs and a small risk of device-related complications. These risks will be reduced with the currently available small injectable ILRs. The use of an ILR could potentially impact patient management, especially when incorporating novel parameters (e. g. third heart sound detection, respiratory rate and respiratory shallowness monitoring), as recently reported in the MultiSENSE study or by combining these continuous parameters with currently available telehomecare strategies [22]. Additional randomised studies are needed in different patient populations to assess whether an ILR could also impact on cost efficiency. However, we anticipate that telemonitoring will increasingly become part of standard heart failure care.
